# Antimicrobial resistance profiles of Shiga toxin-producing *Escherichia coli* O157 and Non-O157 recovered from domestic farm animals in rural communities in Northwestern Mexico

**DOI:** 10.1186/s13756-015-0100-5

**Published:** 2016-01-05

**Authors:** Bianca A. Amézquita-López, Beatriz Quiñones, Marcela Soto-Beltrán, Bertram G. Lee, Jaszemyn C. Yambao, Ofelia Y. Lugo-Melchor, Cristóbal Chaidez

**Affiliations:** Faculty of Chemical and Biological Sciences (FCQB), The Autonomous University of Sinaloa (UAS), Josefa Ortiz de Domínguez y Blvd. de las Américas S/N, Ciudad Universitaria, 80013 Culiacan, Sinaloa México; U.S. Department of Agriculture/Agricultural Research Service, Western Regional Research Center, Produce Safety and Microbiology Research Unit, 800 Buchanan Street, Albany, CA 94710 USA; Centro de Investigación y Asistencia en Tecnología y Diseño del Estado de Jalisco (CIATEJ), Normalistas 800, Colinas de La Normal, 44270 Guadalajara, Jalisco Mexico; Research Center in Food & Development (CIAD), Food Safety National Research Laboratory (LANIIA), Carretera a El Dorado Km5.5, Col. Campo El Diez, 80110 Culiacan, Sinaloa Mexico

**Keywords:** Antimicrobial resistance, Antibiotics, Domestic farm animals, Shiga toxin, *Escherichia coli* O157:H7, *Escherichia coli* non-O157, Mexico

## Abstract

**Background:**

Antimicrobial resistance in Shiga toxin-producing *Escherichia coli* (STEC) O157 and non-O157 is a matter of increasing concern. The aim of the present study was to investigate the antimicrobial resistance profiles of STEC O157 and non-O157 recovered from feces of domestic farm animals in the agricultural Culiacan Valley in Northwestern Mexico.

**Findings:**

All of the examined STEC strains showed susceptibility to five antimicrobials, ceftazidime, ceftriaxone, ciprofloxacin, nalidixic acid, and trimethoprim-sulfamethoxazole. However, resistance to the four antimicrobials, ampicillin, cephalothin, chloramphenicol, and kanamycin was commonly observed. Interestingly, non-susceptibility to cephalothin was predominant among the examined STEC strains, corresponding to 85 % (22/26) of the O157:H7 from cattle, sheep and chicken and 73 % (24/33) of the non-O157 strains from cattle and sheep. Statistical analyses revealed that resistance to ampicillin was significantly correlated to 38 % (10/26) of STEC O157:H7 strains from multiple animal sources. Another significant correlation was found between serotype, source, and antimicrobial resistance; all of the O20:H4 strains, recovered from sheep, were highly resistant to tetracycline. Multidrug resistance profiles were identified in 42 % (22/53) of the non-susceptible STEC strains with clinically-relevant serotypes O8:H9, O75:H8, O146:H21, and O157:H7.

**Conclusions:**

STEC O157 and non-O157 strains, recovered from domestic farm animals in the Culiacan Valley, exhibited resistance to classes of antimicrobials commonly used in Mexico, such as aminoglycosides, tetracyclines, cephalosporins and penicillin but were susceptible to fluoroquinolones, quinolones, and sulfonamides. These findings provide fundamental information that would aid in the surveillance of antimicrobial resistance in an important agricultural region in Northwestern Mexico.

**Electronic supplementary material:**

The online version of this article (doi:10.1186/s13756-015-0100-5) contains supplementary material, which is available to authorized users.

## Introduction

Shiga toxin-producing *Escherichia coli* (STEC) is a foodborne pathogen and causes severe gastroenteritis, hemorrhagic colitis, and the life-threatening hemolytic-uremic syndrome (HUS) in humans [1]. Serotype O157:H7 has been implicated in most outbreaks [1]; however, other non-O157 serotypes have been associated with severe human infections worldwide [1–3]. Recently, several reports have documented a significant increase of antimicrobial resistance in STEC O157:H7 and non-O157:H7 strains [4, 5], and antibiotic resistance of *E. coli* in Mexico has increased over the years [6]. Inappropriate usages of antibiotics for treating human and plant diseases and for promoting food-animal growth are proposed to contribute to antimicrobial resistance among bacteria populations [6–9]. Moreover, the use of antimicrobials to treat STEC infections is controversial since they can induce Shiga toxin (Stx) production, resulting in HUS in humans [10–12]. However, other studies have suggested that if some classes of antimicrobials are administered early during the infection, STEC disease progression to the HUS could be prevented [10, 13, 14].

STEC strains have been recovered from a variety of animals, and cattle are considered the major reservoir for STEC strains [1, 15, 16]. Recent evidence has indicated that small domestic ruminants are also relevant STEC reservoirs [16, 17]. Given that animals act as reservoirs of STEC that could potentially be transmitted to humans, thru direct or indirect contact, or via the food chain, the present study examined antimicrobial susceptibility in STEC O157 and non-O157 strains, recovered from feces of domestic farm animals [16]. The domestic farm animals were raised in small rural communities within the agricultural Culiacan Valley in Northwestern Mexico. The results indicated that STEC O157 and non-O157 strains exhibited resistance to aminoglycosides, tetracyclines, cephalosporins and penicillins, antimicrobials commonly used in Mexico [18–20]. However, all examined STEC strains were susceptible to fluoroquinolones, quinolones, and sulfonamides, agents that can induce Stx production [10, 12, 21]. These findings provide fundamental information that would aid in the surveillance of antimicrobial resistance patterns in an important agricultural region in Northwestern Mexico.

## Materials and methods

### Bacterial strains and growth conditions

A total of 59 STEC O157:H7 and non-O157 strains were isolated from domestic animal feces in small rural farms, near agricultural fields in the Culiacan Valley, Northwestern Mexico [16, 22]. The source, serotype and virulence potential of the tested STEC strains were previously characterized [16, 22]. STEC strains were routinely grown at 37 °C on trypticase soy agar (Bioxon, Mexico City, Mexico) under aerobic conditions.

### Antimicrobial susceptibility testing

The Kirby-Bauer disk diffusion method was performed to test 15 antimicrobials, representing 11 distinct classes (see Additional file 1), which are commonly used in Mexico for animal food production and human infection treatments [18–20]. Inoculums from each STEC strain were grown aerobically in 5 mL Mueller-Hinton (MH) broth (Bioxon, Mexico City, Mexico) and incubated at 37 °C to reach a turbidity equal to a McFarland 0.5 standard, according to guidelines provided by Clinical and Laboratory Standards Institute (CSLI) [23]. MH agar plates were surface inoculated with each STEC culture using sterile cotton swabs, and antimicrobial paper disks (BD Diagnostics, Mexico City, Mexico) were placed on surface of inoculated MH agar plates. After incubation at 37 °C for 16–18 h, the diameter of the zone of microbial growth inhibition around the antimicrobial disk was measured in millimeters. *E. coli* ATCC 25922 (American Type Culture Collection, Manassas, VA) was used as a positive control for antimicrobial susceptibility. The minimum inhibitory concentration (MIC) was then determined according to the interpretive criteria established by CLSI to classify the STEC strains as sensitive, intermediate, or resistant to the tested antimicrobial agent [23].

### Statistical analysis

Statistical differences were determined by performing the Fisher’s exact test with the R Statistical Software (version 3.0.1; R Foundation for Statistical Computing, Vienna, Austria) [24]. A *p*-value ≤ 0.01 was considered statistically significant.

## Results

Susceptibility to all tested 15 antimicrobials was observed in 3 % (1/26) of the O157:H7 cattle strains and 15 % (5/33) non-O157 from cattle and sheep. All of the examined STEC strains showed susceptibility to five antimicrobials, CAZ, CIP, CRO, NAL, and SXT (Tables 1 and 2), and approximately 90 % of the tested STEC strains showed susceptibility to AMC, AMK, CFP, GEN, IPM and TET. By contrast, non-susceptibility to the four antimicrobials, AMP, CEF, CHL, and KAN was commonly observed, and in particular, non-susceptibility to CEF, including intermediate and resistant categories, was predominant among the examined STEC strains, corresponding to 85 % (22/26) of the O157:H7 (Table 1) and 73 % (24/33) of the non-O157 strains (Table 2). Resistance to AMP was significantly correlated to 38 % (10/26) of the O157:H7 strains (*p-*value = 0.0107). A statistically significant correlation was found between serotype, source, and antimicrobial resistance; all of the O20:H4 strains, recovered from sheep, were resistant to TET (*p-*value = 0.0001), accounting for 75 % (3/4) of the TET resistant STEC strains. No other correlation was found for other non-O157 serotypes and antimicrobials tested.

**Table 1:** Antimicrobial MIC Values of STEC O157:H7 strains examined in this study

Serotype	Strain	Source^a^	Antimicrobial MIC Values (μg/mL)														
			AMC	AMK	AMP	CAZ	CEF	CFP	CHL	CIP	CRO	GEN	IPM	KAN	NAL	SXT	TET
O157:H7	RM8744	Cattle	≤8/4	≤ 16	≤ 8	≤ 4	≥ 32^b^	32^c^	16^c^	≤ 1	≤ 1	≤ 4	≤ 1	≤ 16	≤ 16	≤ 2/38	≤ 4
	RM8753	Sheep	≤ 8/4	32^c^	≤ 8	≤ 4	≥ 32^b^	≤ 16	16^c^	≤ 1	≤ 1	≤ 4	≤ 1	32^c^	≤ 16	≤ 2/38	≤ 4
	RM8754	Cattle	≤ 8/4	≤ 16	≤ 8	≤ 4	≤ 8	≤ 16	≤ 8	≤ 1	≤ 1	≤ 4	≤ 1	≤ 16	≤ 16	≤ 2/38	≤ 4
	RM8759	Sheep	≤ 8/4	≤ 16	≤ 8	≤ 4	≥ 32^b^	≤ 16	≤ 8	≤ 1	≤ 1	≤ 4	≤ 1	≤ 16	≤ 16	≤ 2/38	≤ 4
	RM8767	Cattle	≤ 8/4	≤ 16	≤ 8	≤ 4	16^c^	≤ 16	≤ 8	≤ 1	≤ 1	≤ 4	≤ 1	≤ 16	≤ 16	≤ 2/38	≤ 4
	RM8768	Cattle	≤ 8/4	≤ 16	≤ 8	≤ 4	≥ 32^b^	≤ 16	16^c^	≤ 1	≤ 1	≤ 4	≤ 1	≤ 16	≤ 16	≤ 2/38	≤ 4
	RM8769	Cattle	≤ 8/4	≤ 16	≤ 8	≤ 4	≤ 8	≤ 16	16^c^	≤ 1	≤ 1	≤ 4	≤ 1	≤ 16	≤ 16	≤ 2/38	≤ 4
	RM8771	Cattle	≤ 8/4	≤ 16	≤ 8	≤ 4	≤ 8	≤ 16	16^c^	≤ 1	≤ 1	≤ 4	≤ 1	≤ 16	≤ 16	≤ 2/38	≤ 4
	RM8781	Sheep	≤ 8/4	≤ 16	≤ 8	≤ 4	16^c^	≤ 16	≤ 8	≤ 1	≤ 1	≤ 4	≤ 1	32^c^	≤ 16	≤ 2/38	≤ 4
	RM8920	Sheep	≤ 8/4	≤ 16	≤ 8	≤ 4	16^c^	≤ 16	16^c^	≤ 1	≤ 1	≤ 4	≤ 1	≤ 16	≤ 16	≤ 2/38	≤ 4
	RM8921	Sheep	≤ 8/4	≤ 16	≤ 8	≤ 4	≥ 32^b^	≤ 16	16^c^	≤ 1	≤ 1	≤ 4	≤ 1	32^c^	≤ 16	≤ 2/38	≤ 4
	RM8922	Cattle	≤ 8/4	≤ 16	≤ 8	≤ 4	16^c^	≤ 16	≤ 8	≤ 1	≤ 1	≤ 4	≤ 1	≤ 16	≤ 16	≤ 2/38	≤ 4
	RM9450	Sheep	≤ 8/4	≤ 16	≤ 8	≤ 4	≥ 32^b^	≤ 16	≤ 8	≤ 1	≤ 1	≤ 4	≤ 1	≤ 16	≤ 16	≤ 2/38	≤ 4
	RM9451	Sheep	≤ 8/4	≤ 16	≤ 8	≤ 4	16^c^	≤ 16	≥ 32^b^	≤ 1	≤ 1	≤ 4	≤ 1	≥ 64^b^	≤ 16	≤ 2/38	≥ 16^b^
	RM9452	Sheep	≤ 8/4	≤ 16	≤ 8	≤ 4	≥ 32^b^	≤ 16	16^c^	≤ 1	≤ 1	≤ 4	≤ 1	≤ 16	≤ 16	≤ 2/38	≤ 4
	RM9453	Sheep	≤ 8/4	32^c^	≤ 8	≤ 4	≥ 32^b^	≤ 16	16^c^	≤ 1	≤ 1	≤ 4	≤ 1	32^c^	≤ 16	≤ 2/38	≤ 4
	RM9454	Cattle	≤ 8/4	≤ 16	≥ 32^b^	≤ 4	16^c^	≤ 16	≤ 8	≤ 1	≤ 1	≤ 4	≤ 1	≤ 16	≤ 16	≤ 2/38	≤ 4
	RM9455	Cattle	≤ 8/4	≤ 16	≥ 32^b^	≤ 4	16^c^	≤ 16	≤ 8	≤ 1	≤ 1	≤ 4	≤ 1	≤ 16	≤ 16	≤ 2/38	≤ 4
	RM9456	Cattle	≤ 8/4	≤ 16	≥ 32^b^	≤ 4	16^c^	≤ 16	16^c^	≤ 1	≤ 1	≤ 4	≤ 1	32^c^	≤ 16	≤ 2/38	≤ 4
	RM9457	Cattle	≤ 8/4	≤ 16	≥ 32^b^	≤ 4	16^c^	≤ 16	≤ 8	≤ 1	≤ 1	≤ 4	≤ 1	≤ 16	≤ 16	≤ 2/38	≤ 4
	RM9458	Chicken	≤ 8/4	≤ 16	≥ 32^b^	≤ 4	16^c^	≤ 16	≤ 8	≤ 1	≤ 1	≤ 4	≤ 1	≤ 16	≤ 16	≤ 2/38	≤ 4
	RM9459	Chicken	≤ 8/4	≤ 16	≥ 32^b^	≤ 4	16^c^	≤ 16	≤ 8	≤ 1	≤ 1	≤ 4	≤ 1	≤ 16	≤ 16	≤ 2/38	≤ 4
	RM9460	Cattle	≤ 8/4	≤ 16	≥ 32^b^	≤ 4	16^c^	≤ 16	≤ 8	≤ 1	≤ 1	≤ 4	≤ 1	≤ 16	≤ 16	≤ 2/38	≤ 4
	RM9461	Cattle	≤ 8/4	≤ 16	≥ 32^b^	≤ 4	16^c^	≤ 16	≤ 8	≤ 1	≤ 1	≤ 4	≤ 1	≤ 16	≤ 16	≤ 2/38	≤ 4
	RM9462	Cattle	16/8^c^	32^c^	≥ 32^b^	≤ 4	16^c^	≤ 16	≤ 8	≤ 1	≤ 1	≤ 4	≤ 1	32^c^	≤ 16	≤ 2/38	≤ 4
	RM9463	Sheep	≤ 8/4	≤ 16	≥ 32^b^	≤ 4	≤ 8	≤ 16	≤ 8	≤ 1	≤ 1	≤ 4	≤ 1	≤ 16	≤ 16	≤ 2/38	≤ 4

^a^Domestic animal samples were collected from distinct regions in the Culiacan Valley, Sinaloa, Mexico [16]

^b^MIC value in bold indicates resistance to the tested antimicrobial, according to CLSI guidelines [23]

^c^MIC value indicates an intermediate susceptibility to the tested antimicrobial, according to CLSI guidelines [23]

**Table 2:** Antimicrobial MIC Values of STEC non-O157 strains examined in this study

Serotype^a^	Strain	Source^b^	Antimicrobial MIC Values (μg/mL)														
			AMC	AMK	AMP	CAZ	CEF	CFP	CHL	CIP	CRO	GEN	IPM	KAN	NAL	SXT	TET
O8:NT	RM8766	Sheep	≤ 8/4	≤ 16	≤ 8	≤ 4	≤ 8	≤ 16	≤ 8	≤ 1	≤ 1	≤ 4	≤ 1	≤ 16	≤ 16	≤ 2/38	≤ 4
O8:H19	RM8772	Cattle	≤ 8/4	32^d^	≤ 8	≤ 4	16^d^	≤ 16	16^d^	≤ 1	≤ 1	≤ 4	≤ 1	32^d^	≤ 16	≤ 2/38	≤ 4
	RM8773	Cattle	≤ 8/4	≤ 16	≤ 8	≤ 4	≤ 8	≤ 16	≤ 8	≤ 1	≤ 1	≤ 4	≤ 1	≤ 16	≤ 16	≤ 2/38	≤ 4
	RM8774	Cattle	≤ 8/4	≤ 16	≤ 8	≤ 4	16^d^	≤ 16	16^d^	≤ 1	≤ 1	≤ 4	≤ 1	≤ 16	≤ 16	≤ 2/38	≤ 4
	RM8775	Cattle	≤ 8/4	≤ 16	≤ 8	≤ 4	≥ 32^c^	≤ 16	16^d^	≤ 1	≤ 1	≤ 4	≤ 1	32^d^	≤ 16	≤ 2/38	≤ 4
	RM8776	Cattle	≤ 8/4	≤ 16	≤ 8	≤ 4	≥ 32^c^	≤ 16	≥ 32^c^	≤ 1	≤ 1	≤ 4	≤ 1	32^d^	≤ 16	≤ 2/38	≤ 4
O15:NT	RM8747	Cattle	≥ 32/16^c^	≤ 16	≥ 32^c^	≤ 4	≥ 32^c^	≤ 16	16^d^	≤ 1	≤ 1	≤ 4	≤ 1	≤ 16	≤ 16	≤ 2/38	≤ 4
O20:H4	RM8749	Sheep	≤ 8/4	≤ 16	≥ 32^c^	≤ 4	16^d^	≤ 16	≥ 32^c^	≤ 1	≤ 1	≤ 4	≤ 1	≤ 16	≤ 16	≤ 2/38	≥ 16^c^
	RM8750	Sheep	≤ 8/4	≤ 16	16^d^	≤ 4	≥ 32^c^	≤ 16	≤ 8	≤ 1	≤ 1	≤ 4	≤ 1	≤ 16	≤ 16	≤ 2/38	≥ 16^c^
	RM8751	Sheep	≤ 8/4	≤ 16	≤ 8	≤ 4	16^d^	≤ 16	≤ 8	≤ 1	≤ 1	≤ 4	≤ 1	32^d^	≤ 16	≤ 2/38	≥ 16^c^
O73:NT	RM8748	Cattle	≤ 8/4	≤ 16	≤ 8	≤ 4	16^d^	≤ 16	16^d^	≤ 1	≤ 1	≤ 4	≤ 1	≤ 16	≤ 16	≤ 2/38	≤ 4
O73:H4	RM8745	Sheep	≤ 8/4	≤ 16	≤ 8	≤ 4	≥ 32^c^	≤ 16	≤ 8	≤ 1	≤ 1	≤ 4	≤ 1	32^d^	≤ 16	≤ 2/38	≤ 4
	RM8746	Sheep	≤ 8/4	≤ 16	≤ 8	≤ 4	≥ 32^c^	≤ 16	16^d^	≤ 1	≤ 1	≤ 4	≤ 1	32^d^	≤ 16	≤ 2/38	≤ 4
O75:H8	RM8752	Sheep	≤ 8/4	≤ 16	16^d^	≤ 4	≥ 32^c^	≤ 16	≤ 8	≤ 1	≤ 1	≤ 4	≤ 1	32^d^	≤ 16	≤ 2/38	≤ 4
	RM8760	Sheep	≤ 8/4	≤ 16	≤ 8	≤ 4	≤ 8	≤ 16	16^d^	≤ 1	≤ 1	≤ 4	≤ 1	≤ 16	≤ 16	≤ 2/38	≤ 4
	RM8763	Sheep	≤ 8/4	≤ 16	≤ 8	≤ 4	≤ 8	≤ 16	≤ 8	≤ 1	≤ 1	≤ 4	≤ 1	≤ 16	≤ 16	≤ 2/38	≤ 4
	RM8764	Sheep	≤ 8/4	≤ 16	≤ 8	≤ 4	≤ 8	≤ 16	16^d^	≤ 1	≤ 1	≤ 4	≤ 1	≤ 16	≤ 16	≤ 2/38	≤ 4
	RM8765	Sheep	≤ 8/4	≤ 16	≤ 8	≤ 4	16^d^	≤ 16	≤ 8	≤ 1	≤ 1	≤ 4	≤ 1	32^d^	≤ 16	≤ 2/38	≤ 4
	RM8778	Sheep	≤ 8/4	≤ 16	≤ 8	≤ 4	≥ 32^c^	≤ 16	16^d^	≤ 1	≤ 1	≤ 4	≤ 1	32^d^	≤ 16	≤ 2/38	≤ 4
	RM8779	Sheep	≤ 8/4	≤ 16	≤ 8	≤ 4	16^d^	≤ 16	16^d^	≤ 1	≤ 1	≤ 4	≤ 1	≤ 16	≤ 16	≤ 2/38	≤ 4
	RM8780	Sheep	≤ 8/4	≤ 16	≤ 8	≤ 4	≤ 8	≤ 16	16^d^	≤ 1	≤ 1	≤ 4	≤ 1	≤ 16	≤ 16	≤ 2/38	≤ 4
	RM8923	Cattle	≤ 8/4	≤ 16	≤ 8	≤ 4	16^d^	≤ 16	16^d^	≤ 1	≤ 1	≤ 4	≤ 1	32^d^	≤ 16	≤ 2/38	≤ 4
	RM8929	Sheep	≤ 8/4	≤ 16	≤ 8	≤ 4	16^d^	≤ 16	16^d^	≤ 1	≤ 1	≤ 4	≥ 4^c^	32^d^	≤ 16	≤ 2/38	≤ 4
	RM8930	Sheep	≤ 8/4	≤ 16	≤ 8	≤ 4	16^d^	≤ 16	16^d^	≤ 1	≤ 1	≤ 4	≤ 1	32^d^	≤ 16	≤ 2/38	≤ 4
	RM13865	Cattle	≤ 8/4	≤ 16	≥ 32^c^	≤ 4	16^d^	≤ 16	≤ 8	≤ 1	≤ 1	≤ 4	≤ 1	32^d^	≤ 16	≤ 2/38	≤ 4
O111:H8	RM8755	Sheep	≤ 8/4	≤ 16	≤ 8	≤ 4	16^d^	≤ 16	≤ 8	≤ 1	≤ 1	≥ 16^c^	≤ 1	≤ 16	≤ 16	≤ 2/38	≤ 4
	RM8916	Sheep	≤ 8/4	≤ 16	≤ 8	≤ 4	≥ 32^c^	≤ 16	16^d^	≤ 1	≤ 1	≤ 4	≤ 1	≤ 16	≤ 16	≤ 2/38	≤ 4
O146:H8	RM8762	Sheep	≤ 8/4	≤ 16	≤ 8	≤ 4	≤ 8	≤ 16	≤ 8	≤ 1	≤ 1	≤ 4	≤ 1	≤ 16	≤ 16	≤ 2/38	≤ 4
O146:H21	RM8756	Sheep	≤ 8/4	≤ 16	≤ 8	≤ 4	≤ 8	≤ 16	≤ 8	≤ 1	≤ 1	≤ 4	≤ 1	≤ 16	≤ 16	≤ 2/38	≤ 4
	RM8757	Sheep	≤ 8/4	≤ 16	≤ 8	≤ 4	≤ 8	≤ 16	16^d^	≤ 1	≤ 1	≤ 4	≤ 1	≤ 16	≤ 16	≤ 2/38	≤ 4
	RM8758	Sheep	≤ 8/4	≤ 16	≤ 8	≤ 4	16^d^	≤ 16	16^d^	≤ 1	≤ 1	≤ 4	≤ 1	32^d^	≤ 16	≤ 2/38	≤ 4
	RM8761	Sheep	≤ 8/4	≤ 16	≤ 8	≤ 4	16^d^	≤ 16	≤ 8	≤ 1	≤ 1	≤ 4	≤ 1	≤ 16	≤ 16	≤ 2/38	≤ 4
O168:NT	RM8917	Cattle	≤ 8/4	≤ 16	≤ 8	≤ 4	16^d^	≤ 16	≤ 8	≤ 1	≤ 1	≤ 4	≤ 1	32^d^	≤ 16	≤ 2/38	≤ 4

^a^NT, Non-typeable H-antigen

^b^Domestic animal samples were collected from distinct regions in the Culiacan Valley, Sinaloa, Mexico [16]

^c^MIC value indicates resistance to the tested antimicrobial, according to CLSI guidelines [23]

^d^MIC value indicates an intermediate susceptibility to the tested antimicrobial, according to CLSI guidelines [23]

Correlation analysis of non-susceptibility to more than one antimicrobial, belonging to different classes, indicated that 37 % (22/59) of the examined STEC strains exhibited resistance to both the aminoglycoside KAN and to the 1^st^ generation-cephalosporin CEF, resulting in a statistically significant correlation (*p-*value = 0.0001) (Tables 1 and 2). Other observations were that 93 % (14/15) and 80 % (24/30) of the AMP and CHL non-susceptible strains, respectively, also showed non-susceptibility to CEF; however, these associations were not found to be statistically significant. The analyses revealed 19 distinct antimicrobial resistant profiles, and 12 were classified as multidrug resistant profiles (Table 3), indicating non-susceptibility to more than 3 agents in different classes [25]. These multidrug resistance profiles were observed in 42 % (22/53) of the non-susceptible STEC strains with clinically-relevant serotypes O75:H8, O146:H21, O8:H9, and O157:H7 (Table 3). The analysis also revealed that a particular antimicrobial resistance profile was not significantly correlated with animal source or STEC serotype.

**Table 3:** Antimicrobial resistance profiles identified in the STEC O157 and non-O157 from different animal sources

STEC Serotypes (*n*)^a^	Sources^b^	Resistance Profile
O157:H7 (1)	Sheep	AMP
O146:H21 (1), O157:H7 (4)	Sheep Cattle	CEF
O75:H8 (3), O146:H21 (1), O157:H7 (2)	Sheep Cattle	CHL
O157:H7 (7)	Cattle, Chicken	AMP, CEF
O8:H19 (1), O73:NT (1), O75:H8 (1), O111:H8 (1), O157:H7 (3)	Sheep, Cattle	CEF, CHL
O111:H8 (1)	Sheep	CEF, GEN
O157:H7 (1), O73:H4 (1), O75:H8 (1), O168:NT (1)	Sheep, Cattle	CEF, KAN
O75:H8 (2)	Sheep, Cattle	AMP, CEF, KAN
O20:H4 (1)	Sheep	AMP, CEF, TET
O157:H7 (1)	Cattle	CEF, CFP, CHL
O8:H19 (2), O73:H4 (1), O75:H8 (3), O146:H21 (1), O157:H7 (1)	Sheep, Cattle	CEF, CHL, KAN
O20:H4 (1)	Sheep	CEF, KAN, TET
O157:H7 (1)	Cattle	AMP, CEF, CHL, KAN
O20:H4 (1)	Sheep	AMP, CEF, CHL, TET
O75:H8 (1)	Sheep	CEF, CHL, KAN, IPM
O157:H7 (1)	Sheep	CEF, CHL, KAN, TET
O15:NT (1)	Cattle	AMC, AMP, CEF, CHL
O8:H19 (1), O157:H7 (2)	Sheep, Cattle	AMK, CEF, CHL, KAN
O157:H7 (1)	Cattle	AMC, AMK, AMP, CEF, KAN

^a^NT, Non-typeable H-antigen

^b^Domestic animal samples were collected from distinct regions in the Culiacan Valley, Sinaloa, Mexico [16]

## Discussion

Many factors have been proposed to contribute to antimicrobial resistance in enteric bacterial pathogens, such as the inappropriate prescription and use of antibiotics in the public, private, and agricultural sectors [6–9]. Moreover, data from surveillance programs in Mexico have reported an apparent increase in antimicrobial resistance of *E. coli* over the years [6, 18]. In the present study, resistance to antimicrobials, belonging to classes commonly utilized in Mexico [18–20], was investigated in zoonotic STEC. The STEC strains were recovered from domestic farm animals in small rural communities that were adjacent to the agricultural Culiacan Valley, where the primary purpose of raising livestock is for local consumption [16]. The findings of this study provide a better understanding of resistance to antimicrobial agents in an important agricultural region in Mexico and will aid in the development of efficient and targeted intervention strategies.

The results from the present study demonstrated that zoonotic STEC O157 and non-O157, recovered from the Culiacan Valley, were resistant to antimicrobials belonging to classes such as aminoglycosides, beta lactams, carbapenem, cephalosporins, phenicols, and tetracyclines. In particular, resistance to CEF, a 1^st^-generation cephalosporin, was prominently detected in 78 % (46/59) of the tested STEC O157 and non-O157 strains. Interestingly, the present study has demonstrated for the first time a significant correlation for AMP resistance in O157:H7 and TET resistance in O20:H4 zoonotic STEC strains recovered from Northwestern Mexico. In agreement with published findings on STECs recovered from foods in this geographical region [18], susceptibility was observed for sulfonamides, quinolones and fluoroquinolones in the recovered STEC strains. These agents have been found to induce Stx production in STEC strains [10, 21], potentially increasing the risk of HUS. However, all STEC strains were susceptible to the 3^rd^-generation cephalosporin CRO, which does not promote Stx production [21].

Classification of multidrug-resistance, based on recently published criteria [25], was observed in 42 % (22/53) of the non-susceptible STEC strains, harboring serotypes associated with human illness [2]. Multidrug resistance profiles, described in the present study, included classes of antimicrobials commonly used in Mexico, such as aminoglycosides, tetracyclines, cephalosporins, and penicillins [18–20], and these findings highlight the need for surveillance of the antimicrobial resistance patterns in enteric bacterial pathogens. Future work is aimed at further dissecting the genetic elements contributing to the acquisition and dissemination of the antimicrobial resistance genes in STEC strains recovered from agricultural regions in Northwestern Mexico.

## Additional file

Additional file 1:
**Antimicrobial agents used in the present study.** (DOCX 14 kb)

## Abbreviations

AMC: Amoxicillin – clavulanic acid
AMK: Amikacin
AMP: Ampicillin
CAZ: Ceftazidime
CEF: Cephalothin
CFP: Cefoperazone
CHL: Chloramphenicol
CIP: Ciprofloxacin
CLSI: Clinical and Laboratory Standards Institute
CRO: Ceftriaxone
GEN: Gentamicin
HUS: Hemolytic uremic syndrome
IPM: Imipenem
KAN: Kanamycin
MDR: Multi-drug resistant
MH: Mueller-Hinton
MIC: Minimum inhibitory concentration
NAL: Nalidixic acid
STEC: Shiga toxin-producing *Escherichia coli*
Stx: Shiga toxin
SXT: Trimethoprim-sulfamethoxazole
TET: Tetracycline
