# Supplementary material for: Antimicrobial resistance profiles of Shiga toxin-producing Escherichia coli O157 and Non-O157 recovered from domestic farm animals in rural communities in Northwestern Mexico
Source: Antimicrob Resist Infect Control. 2016 Jan 5;5:1. doi: 10.1186/s13756-015-0100-5 (PMC4700648; doi:10.1186/s13756-015-0100-5)
Supplement: Additional file 1: — Antimicrobial agents used in the present study. (DOCX 14 kb) [file 13756_2015_100_MOESM1_ESM.docx]

**Additional File 1. Antimicrobial agents used in the present study.**

| Class of Antimicrobial | Antimicrobial Agent | Abbreviation | Concentration (µg) | MIC Interpretive Criteria (µg/mL)^a^ | | |
| --- | --- | --- | --- | --- | --- | --- |
|  |  |  |  | Sensitive | Intermediate | Resistant |
| Aminoglycosides | Amikacin | AMK | 30 | ≤ 16 | 32 | ≥ 64 |
|  | Gentamicin | GEN | 10 | ≤ 4 | 8 | ≥ 16 |
|  | Kanamycin | KAN | 30 | ≤ 16 | 32 | ≥ 64 |
| β-Lactamase Inhibitors | Amoxicillin - Clavulanic Acid | AMC | 20/10 | ≤ 8/4 | 16/8 | ≥ 32/16 |
| Carbapenems | Imipenem | IPM | 10 | ≤ 1 | 2 | ≥ 4 |
| Cephalosporins  (1^st^ generation) | Cephalothin | CEF | 30 | ≤ 8 | 16 | ≥ 16 |
| Cephalosporins  (3^rd^ generation) | Cefoperazone | CFP | 75 | ≤ 16 | 32 | ≥ 64 |
|  | Ceftazidime | CAZ | 30 | ≤ 4 | 8 | ≥ 16 |
|  | Ceftriaxone | CRO | 30 | ≤ 1 | 2 | ≥ 4 |
| Fluoroquinolones | Ciprofloxacin | CIP | 5 | ≤ 1 | 2 | ≥ 4 |
| Penicillins | Ampicillin | AMP | 10 | ≤ 8 | 16 | ≥ 32 |
| Phenicols | Chloramphenicol | CHL | 30 | ≤ 8 | 16 | ≥ 32 |
| Quinolones | Nalidixic Acid | NAL | 30 | ≤ 16 | - | ≥ 32 |
| Sulfonamides | Trimethoprim-Sulfamethoxazole | SXT | 23.75/1.25 | ≤ 2/38 | - | ≥ 4/76 |
| Tetracyclines | Tetracycline | TET | 30 | ≤ 4 | 8 | ≥ 16 |

^a^MIC interpreted criteria according to guidelines provided by the Clinical & Laboratory Standards Institute [[23](#_ENREF_23)].
